# Impact of body mass index on real-world outcomes of rivaroxaban treatment in Japanese patients with non-valvular atrial fibrillation

**DOI:** 10.1007/s00380-020-01587-z

**Published:** 2020-04-06

**Authors:** Yuji Murakawa, Takanori Ikeda, Satoshi Ogawa, Takanari Kitazono, Jyoji Nakagawara, Kazuo Minematsu, Susumu Miyamoto, Yasuhiro Hayashi, Yoko Kidani, Yutaka Okayama, Toshiyuki Sunaya, Shoichiro Sato, Satoshi Yamanaka

**Affiliations:** 1 The 4th Department of Internal Medicine, Teikyo University School of Medicine, Mizonokuchi Hospital, 5-1-1, Futago, Takatsu-ku, Kawasaki, Kanagawa 213-8507 Japan; 2 Department of Cardiovascular Medicine, Toho University Graduate School of Medicine, Tokyo, Japan; 3 International University of Health and Welfare Mita Hospital, Tokyo, Japan; 4 Department of Medicine and Clinical Science, Graduate School of Medical Sciences, Kyushu University, Fukuoka, Japan; 5 Osaka Namba Clinic, Osaka, Japan; 6 National Cerebral and Cardiovascular Center, Suita, Osaka Japan; 7 Iseikai Medical Corporation, Osaka, Japan; 8 Department of Neurosurgery, Kyoto University Graduate School of Medicine, Kyoto, Japan; 9 Medical Affairs Thrombosis, Medical Affairs, Bayer Yakuhin, Ltd., Osaka, Japan; 10 Pharmacovigilance Monitoring and Medical Governance, Medical Affairs, Bayer Yakuhin, Ltd., Osaka, Japan; 11 Research and Development Japan/Data Sciences and Analytics/Statistics and Data Insights, Bayer Yakuhin, Ltd., Osaka, Japan

**Keywords:** Atrial fibrillation, Body mass index, Stroke, Rivaroxaban

## Abstract

This sub-analysis of the XAPASS, a prospective, single-arm, observational study, aimed to evaluate relationships between body mass index (BMI) and safety (major bleeding and all-cause mortality) and effectiveness [stroke/non-central nervous system (non-CNS) systemic embolism (SE)/myocardial infarction (MI)] outcomes in Japanese patients with non-valvular atrial fibrillation (NVAF) receiving rivaroxaban. Patients were categorized according to BMI (kg/m^2^) as underweight (< 18.5), normal weight (18.5 to < 25), overweight (25 to < 30), or obese (≥ 30). In total, 9578 patients with NVAF completed the 1-year follow-up and were evaluated; of these, 7618 patients had baseline BMI data. Overall, 542 (5.7%), 4410 (46.0%), 2167 (22.6%), and 499 (5.2%) patients were underweight, normal weight, overweight, and obese, respectively. Multivariable Cox regression analysis demonstrated that none of the BMI categories were independent predictors of major bleeding whereas being underweight was independently associated with increased all-cause mortality [hazard ratio (HR) 3.56, 95% confidence interval (CI) 2.40–5.26, *p* < 0.001]. The incidence of stroke/non-CNS SE/MI was higher in patients who were underweight than in those of normal weight (HR 2.11, 95% CI 1.20–3.70, *p* = 0.009). However, in multivariable analyses, being underweight was not identified as an independent predictor of stroke/non-CNS SE/MI (HR 1.64, 95% CI 0.90–2.99, *p* = 0.104). In conclusion, the high incidence of thromboembolic events and all-cause mortality in patients who were underweight highlights that thorough evaluation of disease status and comorbidities may be required in this population.

**Electronic supplementary material:**

The online version of this article (10.1007/s00380-020-01587-z) contains supplementary material, which is available to authorized users.

## Introduction

Direct oral anticoagulants (DOACs) such as rivaroxaban offer an alternative to vitamin K antagonists as anticoagulant treatments for stroke prevention in non-valvular atrial fibrillation (NVAF) [1]. Over the past decade, there has been a growing interest in the relationship between body mass index (BMI) and clinical outcomes in patients with atrial fibrillation (AF). Although obesity is a risk factor for AF, some studies have demonstrated that among patients with AF, mortality is lower in those with obesity than in those of normal weight, a phenomenon known as the obesity paradox [2–4]. However, the impact of being underweight on the incidence of thromboembolism and bleeding outcomes has been difficult to assess because patients who are underweight are often underrepresented in global or Western clinical trials and observational studies. Moreover, many trials do not consider differences in body composition; for example, Western patients tend to be taller and have higher body weight than Asian patients. A recent meta-analysis, which included Asian patients, demonstrated that being underweight was associated with an increased risk of stroke or systemic embolism (SE) [5]; however, other studies showed no associations between being underweight and the incidence of thromboembolism [6, 7]. These inconsistent data suggest a need to further examine the impact of BMI on outcomes in patients being treated for stroke prevention in AF. In the present sub-analysis of the Xarelto Post-Authorization Safety & Effectiveness Study in Japanese Patients with Atrial Fibrillation (XAPASS), we examined the relationship between BMI and clinical outcomes in Japanese patients with NVAF using the XAPASS 1-year follow-up data.

## Materials and methods

### Study design

The XAPASS (Clinicaltrials.gov: NCT01582737) is a real-world, prospective, open-label, single-arm, observational, post-authorization cohort study conducted in Japan. The study design has been described previously [8]. Briefly, the standard observation period for each patient is 2 years with data collection at 6 months, 1 year, and 2 years after the initiation of rivaroxaban treatment. After the completion of the standard observation period, follow-up investigations will be conducted for a maximum of 5 years. The study was approved by the Ministry of Health, Labour, and Welfare in Japan and was carried out in accordance with the standards for Good Post-marketing Study Practice provided by this ministry. Individual consent and institutional approval of ethical standards in accordance with the Declaration of Helsinki are not necessary in activities and research for the safety surveillance, such as signal detection and prospective cohort studies.

### Patients

In total, 11,308 Japanese patients with NVAF were enrolled in the XAPASS between April 2012 and June 2014. The current sub-analysis included 9578 patients who had completed at least 11 months of the 1-year follow-up, discontinued rivaroxaban treatment within 1 year, or were lost to follow-up within 1 year [9]. The patients were divided into four BMI (kg/m^2^) categories according to the World Health Organization technical report series: underweight (< 18.5), normal weight (18.5 to < 25), overweight (25 to < 30), and obese (≥ 30) [10].

### Treatment

Patients received oral rivaroxaban at a dosage of either 15 mg once daily (o.d.) or 10 mg o.d., at the discretion of the treating physician. These dosages are approved in Japan for patients with creatinine clearance (CrCl) ≥ 50 and < 50 ml/min, respectively.

### Study outcomes

The primary safety outcome was any bleeding. Major bleeding and intracranial hemorrhage were recorded as the components. Major bleeding was defined according to the International Society on Thrombosis and Haemostasis criteria [11]. The primary effectiveness outcome was a composite of stroke (hemorrhagic or ischemic), non-central nervous system (non-CNS) SE, and myocardial infarction (MI). All the outcomes were defined previously [8, 9]. Stroke and ischemic stroke were recorded as individual outcomes. Transient ischemic attack (TIA) was not included in the stroke endpoint.

### Statistical analysis

Patient characteristics at baseline were summarized by frequencies and percentages for categorical data. Survival curves were estimated by the Kaplan–Meier method. Univariable and multivariable Cox regression analyses were performed to estimate hazard ratios (HRs) of outcomes within the four BMI categories. The following variables at the study enrollment were included as explanatory variables: age, sex, CrCl, initial dose, hypertension, diabetes mellitus, congestive heart failure, prior ischemic stroke/TIA, vascular disease, hepatic dysfunction, and oral antiplatelet use considering medical interest, multicollinearity, and data availability. All statistical analyses were performed using SAS version 9.2 or higher (SAS Institute Inc., Cary, NC).

## Results

### Patients

In total, 9578 patients with NVAF completed the 1-year follow-up and were evaluated and 7618 patients had baseline BMI data. Overall, 542 (5.7%), 4,410 (46.0%), 2,167 (22.6%), and 499 (5.2%) patients were underweight, normal weight, overweight, and obese, respectively; demographic characteristics varied across the BMI categories (Table 1). Compared with patients of normal weight, a numerically higher proportion of those categorized as underweight were female or ≥ 75 years of age. Among those who were underweight, normal weight, overweight, and obese, 62.9%, 29.1%, 11.6%, and 6.2% had CrCl < 50 ml/min, respectively. The proportion of patients with comorbid hypertension and diabetes was highest among patients with obesity and lowest among those who were underweight. CHADS_2,_ CHA_2_DS_2_-VASc, and modified HAS-BLED scores were similar across BMI groups (Table 1).

**Table 1:** Baseline characteristics

	BMI (kg/m^2^)			
	Underweight (< 18.5) (*n* = 542)	Normal (18.5 to < 25) (*n* = 4410)	Overweight (25 to < 30) (*n* = 2167)	Obese (≥ 30) (*n* = 499)
BMI, mean (± SD), kg/m^2^	17.1 (± 1.1)	22.2 (± 1.7)	26.9 (± 1.4)	33.4 (± 3.8)
Age, years				
Mean (± SD)	78.1 (± 9.0)	73.6 (± 9.6)	71.3 (± 9.6)	69.5 (± 11.0)
< 75	166 (30.6)	2209 (50.1)	1274 (58.8)	305 (61.1)
≥ 75	376 (69.4)	2201 (49.9)	893 (41.2)	194 (38.9)
Female sex	287 (53.0)	1638 (37.1)	704 (32.5)	221 (44.3)
CrCl, ml/min				
Mean (± SD)	47.0 (± 18.8)	63.0 (± 28.5)	77.5 (± 25.6)	97.9 (± 36.4)
< 50	341 (62.9)	1284 (29.1)	252 (11.6)	31 (6.2)
≥ 50	195 (36.0)	3091 (70.1)	1893 (87.4)	461 (92.4)
Unknown	6 (1.1)	35 (0.8)	22 (1.0)	7 (1.4)
Comorbidities				
Hypertension	323 (59.6)	3195 (72.4)	1,804 (83.2)	437 (87.6)
Diabetes mellitus	81 (14.9)	869 (19.7)	596 (27.5)	197 (39.5)
Prior ischemic stroke/TIA	153 (28.2)	1032 (23.4)	421 (19.4)	100 (20.0)
Congestive heart failure	162 (29.9)	1099 (24.9)	529 (24.4)	131 (26.3)
Hepatic dysfunction	31 (5.7)	254 (5.8)	148 (6.8)	50 (10.0)
Type of AF				
Paroxysmal	197 (36.3)	1546 (35.1)	723 (33.4)	143 (28.7)
Persistent	200 (36.9)	1582 (35.9)	803 (37.1)	192 (38.5)
Permanent	117 (21.6)	1041 (23.6)	552 (25.5)	141 (28.3)
Other	0 (0)	9 (0.2)	9 (0.4)	0 (0)
Unknown	28 (5.2)	232 (5.3)	80 (3.7)	23 (4.6)
CHADS_2_ score				
Mean (± SD)	2.3 (± 1.4)	2.2 (± 1.3)	2.2 (± 1.3)	2.3 (± 1.2)
0	58 (10.7)	450 (10.2)	137 (6.3)	16 (3.2)
1	97 (17.9)	1,031 (23.4)	606 (28.0)	121 (24.3)
2	158 (29.2)	1,330 (30.2)	682 (31.5)	156 (31.3)
3	113 (20.8)	848 (19.2)	389 (18.0)	121 (24.2)
4	77 (14.2)	516 (11.7)	236 (10.9)	58 (11.6)
5	34 (6.3)	197 (4.5)	89 (4.1)	23 (4.6)
6	5 (0.9)	38 (0.9)	28 (1.3)	4 (0.8)
CHA_2_DS_2_-VASc score				
Mean (± SD)	3.8 (± 1.6)	3.4 (± 1.6)	3.3 (± 1.6)	3.5 (± 1.6)
0	7 (1.3)	123 (2.8)	56 (2.6)	7 (1.4)
1	39 (7.2)	448 (10.2)	220 (10.2)	36 (7.2)
2	62 (11.4)	698 (15.8)	439 (20.3)	92 (18.4)
3	123 (22.7)	1,050 (23.8)	515 (23.8)	115 (23.0)
4	130 (24.0)	1,013 (23.0)	454 (21.0)	114 (22.8)
5	102 (18.8)	629 (14.3)	270 (12.5)	81 (16.2)
6	51 (9.4)	319 (7.2)	148 (6.8)	37 (7.4)
7	28 (5.2)	109 (2.5)	49 (2.3)	14 (2.8)
8	0 (0)	20 (0.5)	16 (0.7)	3 (0.6)
9	0 (0)	1 (< 0.1)	0 (0)	0 (0)
Modified HAS-BLED score^a^				
Mean (± SD)	1.7 (± 0.9)	1.5 (± 1.0)	1.4 (± 1.0)	1.5 (± 1.0)
0	31 (5.7)	528 (12.0)	332 (15.3)	77 (15.4)
1	215 (39.7)	1,776 (40.3)	905 (41.8)	204 (40.9)
2	191 (35.2)	1,431 (32.4)	650 (30.0)	145 (29.1)
3	89 (16.4)	543 (12.3)	226 (10.4)	56 (11.2)
4	12 (2.2)	120 (2.7)	46 (2.1)	16 (3.2)
5	4 (0.7)	12 (0.3)	6 (0.3)	1 (0.2)
6	0 (0)	0 (0)	1 (< 0.1)	0 (0)
7	0 (0)	0 (0)	0 (0)	0 (0)
8	0 (0)	0 (0)	0 (0)	0 (0)
Visit classification				
In-hospital	172 (31.7)	768 (17.4)	271 (12.5)	66 (13.2)
Outpatient	370 (68.3)	3,642 (82.6)	1,896 (87.5)	433 (86.8)

Data are presented as *n* (%) unless otherwise indicated

*AF* atrial fibrillation, *BMI* body mass index, *CHADS*_*2*_ Congestive heart failure, Hypertension, Age ≥ 75 years, Diabetes, Prior stroke or TIA, *CHA*_*2*_*DS*_*2*_*-VASc* Congestive heart failure, Hypertension, Age ≥ 75 years, Age 65–74 years, Diabetes mellitus, Prior stroke or TIA or Thromboembolism, Sex, Vascular disease (e.g. peripheral artery disease, myocardial infarction or aortic plaque), *CrCl* creatinine clearance, HAS-*BLED* Hypertension, Abnormal renal and liver function, Stroke, Bleeding, Labile INR, Elderly, Drugs or alcohol, *SD* standard deviation, *TIA* transient ischemic attack

^a^Maximum score is 8 because of the exclusion of the factor "labile international normalized ratio” from the HAS-BLED score

### Treatment period and rivaroxaban dosage

The mean (± standard deviation) treatment duration was 259 (± 140), 301 (± 117), 307 (± 114), and 306 (± 115) days in patients who were underweight, normal weight, overweight, and obese, respectively. Among those with CrCl < 50 ml/min, 8.1% (44/542), 3.0% (134/4410), 1.0% (22/2167), and 0.6% (3/499) in the underweight, normal weight, overweight, and obese group, respectively, received rivaroxaban 15 mg o.d. (overdose) (Supplementary Fig. 1). Among those with CrCl ≥ 50 ml/min, 17.9% (97/542), 23.9% (1054/4410), 31.0% (672/2167), and 32.5% (162/499) in the underweight, normal weight, overweight, and obese group, respectively, received rivaroxaban 10 mg o.d. (underdose) (Supplementary Fig. 1).

### Safety and effectiveness outcomes

**Fig. 1:**
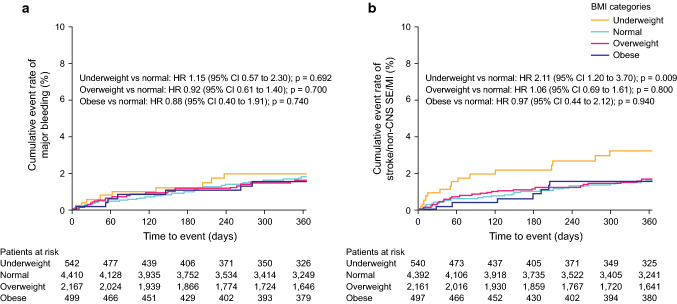
Kaplan–Meier curves for the cumulative event rate of **a** major bleeding and **b** stroke/non-CNS SE/MI among four BMI categories*. *BMI* body mass index, *CI* confidence interval, *CNS* central nervous system, *HR* hazard ratio, *MI* myocardial infarction, *SE* systemic embolism. *BMI categories (kg/m^2^): underweight, < 18.5; normal, 18.5 to < 25; overweight, 25 to < 30; and obese, ≥ 30

Safety and effectiveness outcome event rates are shown in Table 2 and Supplementary Table 1. Major bleeding event rates per 100 patient-years were 1.87 in the normal weight (reference) group, 2.19 in the underweight group, 1.71 in the overweight group, and 1.63 in the obese group (Table 2). The cumulative incidences of major bleeding were similar across all groups when normal BMI was used as the reference [HR 1.15, 95% confidence interval (CI) 0.57–2.30, *p* = 0.692 for patients who were underweight; HR 0.92, 95% CI 0.61–1.40, *p* = 0.700 for patients who were overweight; HR 0.88, 95% CI 0.40–1.91, *p* = 0.740 for patients who were obese] (Fig. 1a and Table 3). Multivariable analysis demonstrated no independent associations between BMI categories and major bleeding (Table 3).

**Table 2:** Incidences of safety and effectiveness outcomes

	BMI (kg/m^2^)			
	Underweight (< 18.5)	Normal (18.5 to < 25)	Overweight (25 to < 30)	Obese (≥ 30)
	Incidence, events per 100 patient-years (95% CI)			
Safety outcome	(*n* = 542)	(*n* = 4410)	(*n* = 2167)	(*n* = 499)
Any bleeding	11.72 (8.33–15.11)	8.42 (7.47–9.36)	7.04 (5.82–8.26)	5.47 (3.23–7.70)
Major bleeding	2.19 (0.76–3.62)	1.87 (1.43–2.30)	1.71 (1.12–2.31)	1.63 (0.42–2.84)
Fatal	0.24 (0.00–0.72)	0.11 (0.00–0.21)	0.32 (0.06–0.58)	0 (0)
Critical organ bleeding	0.73 (0.00–1.55)	0.85 (0.56–1.14)	0.96 (0.52–1.41)	0 (0)
Intracranial hemorrhage	0.73 (0.00–1.55)	0.77 (0.49–1.05)	0.80 (0.40–1.21)	0 (0)
Hemoglobin decrease ≥ 2 g/dl	0.73 (0.00–1.55)	0.69 (0.43–0.96)	0.53 (0.20–0.86)	1.16 (0.14–2.18)
Transfusion of ≥ 2 units of packed RBC or whole blood	0.48 (0.00–1.16)	0.21 (0.07–0.36)	0.16 (0.00–0.34)	0.23 (0.00–0.69)
All-cause mortality	10.66 (7.51–13.81)	1.91 (1.47–2.35)	1.76 (1.16–2.36)	0.93 (0.02–1.84)

Effectiveness outcome	(*n* = 540)	(*n* = 4392)	(*n* = 2161)	(*n* = 497)
Stroke/non-CNS SE/MI	3.67 (1.81–5.53)	1.69 (1.27–2.10)	1.77 (1.17–2.38)	1.63 (0.42–2.84)
Stroke	2.94 (1.28–4.60)	1.55 (1.15–1.95)	1.61 (1.04–2.19)	1.16 (0.14–2.18)
Ischemic stroke	2.20 (0.76–3.64)	1.07 (0.74–1.40)	1.07 (0.60–1.54)	1.16 (0.14–2.18)

*BMI* body mass index, *CI* confidence interval, *CNS* central nervous system, *MI* myocardial infarction, *RBC* red blood cell, *SE* systemic embolism

**Table 3:** Risk of major bleeding, all-cause mortality, and stroke/non-CNS SE/MI

Variable^a^	Number of events	Proportion (%)	Univariable analysis			Multivariable analysis^b^		
			HR	95% CI	*p* value	HR	95% CI	*p* value
Major bleeding								
Underweight	9/542	1.66	1.15	(0.57–2.30)	0.692	0.97	(0.48–1.98)	0.939
Normal	70/4,410	1.59	1.00	Reference		1.00	Reference	
Overweight	32/2,167	1.48	0.92	(0.61–1.40)	0.700	0.97	(0.63–1.50)	0.906
Obese	7/499	1.40	0.88	(0.40–1.91)	0.740	0.89	(0.40–1.97)	0.772
All-cause mortality								
Underweight	44/542	8.12	5.51	(3.78–8.01)	< 0.001	3.56	(2.40–5.26)	< 0.001
Normal	72/4,410	1.63	1.00	Reference		1.00	Reference	
Overweight	33/2,167	1.52	0.92	(0.61–1.39)	0.705	1.19	(0.77–1.83)	0.427
Obese	4/499	0.80	0.49	(0.18–1.33)	0.161	0.74	(0.27–2.05)	0.562
Stroke/non-CNS SE/MI								
Underweight	15/540	2.78	2.11	(1.20–3.70)	0.009	1.64	(0.90–2.99)	0.104
Normal	63/4,392	1.43	1.00	Reference		1.00	Reference	
Overweight	33/2,161	1.53	1.06	(0.69–1.61)	0.800	1.19	(0.76–1.84)	0.447
Obese	7/497	1.41	0.97	(0.44–2.12)	0.940	1.15	(0.52–2.57)	0.727

*BMI* body mass index, *CHF* congestive heart failure, *CI* confidence interval, *CNS* central nervous system, *CrCl* creatinine clearance, *HR* hazard ratio, *MI* myocardial infarction, *SE* systemic embolism, *TIA* transient ischemic attack

^a^BMI categories (kg/m^2^): underweight, < 18.5; normal, 18.5 to < 25; overweight, 25 to < 30; and obese, ≥ 30

^b^Adjusted for age (≥ 75/ < 75 years), sex (female/male), CrCl (< 50/ ≥ 50 ml/min), initial dose (10 mg/15 mg), hypertension (yes/no), diabetes mellitus (yes/no), CHF (yes/no), prior ischemic stroke/TIA (yes/no), vascular disease (yes/no), hepatic dysfunction (yes/no), and oral antiplatelet use (yes/no)

**Table 4:** Adverse events leading to all-cause mortality

	BMI (kg/m^2^)			
	Underweight (< 18.5) (*n* = 542)	Normal (18.5 to < 25) (*n* = 4410)	Overweigh (25 to < 30) (*n* = 2167)	Obese (≥ 30) (*n* = 499)
Adverse events	44	72	33	4
MedDRA System Organ Class^a^				
Cardiac disorders^b^	12 (2.21)	13 (0.29)	8 (0.37)	1 (0.20)
Cancer^b^	9 (1.66)	15 (0.34)	5 (0.23)	1 (0.20)
Respiratory disorders^b^	8 (1.48)	12 (0.27)	5 (0.23)	0 (0)
Infections^b^	5 (0.92)	14 (0.32)	2 (0.09)	2 (0.40)
General and whole-body disorders	3 (0.55)	6 (0.14)	5 (0.23)	0 (0)
Renal disorders	2 (0.37)	1 (0.02)	0 (0)	0 (0)
Nervous system disorders	1 (0.18)	7 (0.16)	5 (0.23)	0 (0)
Metabolism and nutrition disorders	1 (0.18)	4 (0.09)	1 (0.05)	0 (0)
Injury, poisoning, and procedural complications	1 (0.18)	3 (0.07)	1 (0.05)	0 (0)
Vascular disorders	0 (0)	0 (0)	2 (0.09)	0 (0)

Data are presented as *n* (%). MedDRA terms with ≥ 2 events in either BMI categories are shown

*BMI* body mass index, *MedDRA* Medical Dictionary for Regulatory Activities

^a^Based on MedDRA version 20.0

^b^The terms used in MedDRA are as follows: cancer, neoplasms benign, malignant, and unspecified (including cysts and polyps); infections, infections and infestations; respiratory disorders, respiratory, thoracic, and mediastinal disorders

All-cause mortality per 100 patient-years was 1.91 in the normal weight (reference) group, 10.66 in the underweight group, 1.76 in the overweight group, and 0.93 in the obese group (Table 2). The univariable analysis demonstrated that, compared with patients of normal weight, all-cause mortality was significantly higher in patients who were underweight (HR 5.51, 95% CI 3.78–8.01, *p* < 0.001) and similar in those who were overweight (HR 0.92, 95% CI 0.61–1.39, *p* = 0.705) or obese (HR 0.49, 95% CI 0.18–1.33, *p* = 0.161) (Table 3). Multivariable analyses with normal weight as the reference demonstrated an independent association between being underweight and all-cause mortality (HR 3.56, 95% CI 2.40–5.26, *p* < 0.001) (Table 3). Being overweight (HR 1.19, 95% CI 0.77–1.83, *p* = 0.427) or obese (HR 0.74, 95% CI 0.27–2.05, *p* = 0.562) was not associated with increased all-cause mortality (Table 3).

The incidence per 100 patient-years of stroke/non-CNS SE/MI was 1.69 in the normal weight (reference) group, 3.67 in the underweight group, 1.77 in the overweight group, and 1.63 in the obese group (Table 2). Compared with patients of normal weight, the cumulative incidence of stroke/non-CNS SE/MI was significantly higher in patients who were underweight (HR 2.11, 95% CI 1.20–3.70, *p* = 0.009) and similar in those who were overweight (HR 1.06, 95% CI 0.69–1.61, *p* = 0.800) or obese (HR 0.97, 95% CI 0.44–2.12, *p* = 0.940) (Fig. 1b and Table 3). Multivariable analysis with normal BMI as the reference identified no independent associations between any of the BMI categories and the incidence of stroke/non-CNS SE/MI (Table 3).

### Adverse events leading to all-cause mortality

Overall, 8.1%, 1.6%, 1.5%, and 0.8% of patients in the underweight, normal weight, overweight, and obese group, respectively, experienced an adverse event that led to death (all-cause mortality) (Supplementary Table 1). In particular, 0.37%, 0.18%, 0.37%, and 0% of patients in the underweight, normal weight, overweight, and obese group, respectively, died as a result of adverse drug reactions (Supplementary Table 1). Compared with those in the normal, overweight, and obese categories, there was a trend towards a greater proportion of patients who were underweight dying from cardiac disorders, cancer, and respiratory disorders (Table 4).

## Discussion

The key findings of this study in Japanese patients with NVAF receiving rivaroxaban can be summarized as follows: first, baseline characteristics varied across BMI categories; second, the incidences of stroke/non-CNS SE/MI and all-cause mortality were significantly higher in those who were underweight than in those of normal weight; third, none of the BMI categories emerged as an independent predictor of major bleeding or stroke/non-CNS SE/MI; fourth, in multivariable analyses, being underweight was found to be an independent predictor of all-cause mortality.

Limited conclusions could be drawn from previous global studies investigating the impact of BMI on outcomes in patients with AF receiving anticoagulants because very few patients included in these analyses were underweight [7, 12–14]. The efficacy and safety of rivaroxaban in patients with NVAF were established in the global Rivaroxaban Once Daily Oral Direct Factor Xa Inhibition Compared with Vitamin K Antagonism for Prevention of Stroke and Embolism Trial in Atrial Fibrillation (ROCKET AF) and Japan-specific J-ROCKET AF phase 3 studies [15, 16]. In a post hoc analysis of the ROCKET AF study, high BMI was associated with reduced stroke risk, whereas bleeding risk was similar across BMI categories (18.50–24.99, 25.00–29.99, and ≥ 30 kg/m^2^) [17]. These data suggest that BMI is associated with efficacy and safety outcomes in patients receiving rivaroxaban for stroke prevention in NVAF; however, there were limited data in patients who are underweight (i.e. BMI < 18.5 kg/m^2^), because these patients were excluded from the post hoc analysis and constituted only ~ 1% of the ROCKET AF population [15]. Although J-ROCKET AF may have included a greater proportion of patients who were underweight than ROCKET AF, sample sizes were too small to evaluate the influence of BMI on clinical outcomes. A meta-analysis of studies that included Asian patients with AF concluded that being underweight was associated with an increased risk of stroke or SE, cardiovascular death, and all-cause death, whereas in all patients with AF, being overweight or obese was not associated with increased risks of these outcomes, data that support the existence of the obesity paradox [5]. However, this study did not evaluate potential associations between BMI and bleeding outcomes. Data from another recent meta-analysis that included Western and Asian patients also supported the existence of the obesity paradox; however, this study included patients with body weight < 60 kg in the BMI < 18.5 kg/m^2^ group if BMI information was not available, which may not properly reflect differences in body composition between Western and Asian patients [18]. Previous studies in Japanese patients with AF have suggested that those who were underweight had worse prognosis than those of normal weight, but these studies were conducted during the warfarin era [6, 19].

The XAPASS was conducted to evaluate the real-world safety and effectiveness of rivaroxaban in patients with NVAF [8]. The 1-year follow-up results in 9578 patients demonstrated low incidences of bleeding and thromboembolic events, suggesting that rivaroxaban is safe and effective for stroke prevention in daily clinical practice [9]. Compared with previous studies, such as the ROCKET AF post hoc analysis, the present sub-analysis of the XAPASS included a population with more diverse demographic characteristics and a greater proportion of patients were underweight (BMI < 18.5 kg/m^2^). The XAPASS dataset was therefore more suitable for the evaluation of potential associations between BMI and clinical outcomes in patients receiving rivaroxaban than the ROCKET AF post hoc analysis.

Results from the present XAPASS sub-analysis in Japanese patients with NVAF receiving rivaroxaban showed that BMI was not independently associated with the incidence of major bleeding even though the incidence of major bleeding in patients who were underweight was numerically higher than in those of normal weight. Similar results were reported in the J-RHYTHM registry sub-analysis, which showed a trend towards increasing rates of major hemorrhage with decreasing BMI, but that being underweight was not associated with a significantly increased risk of hemorrhage compared with patients of normal weight [6]. However, Park et al*.* demonstrated that patients who were underweight had a significantly increased risk of major bleeding compared with those of normal weight [7]. These inconsistencies may be due to differences in study design (e.g. observational or retrospective), sample size, and class of oral anticoagulant administered (e.g. warfarin or DOAC).

Data from the present study demonstrated no significant association between BMI and the incidence of thromboembolism, which is consistent with results from previous studies [6, 7]. However, a significantly higher incidence of stroke/non-CNS SE/MI was observed in patients who were underweight than in those of normal weight, which may be partially explained by differences in demographic characteristics between BMI groups. For example, patients who were underweight tended to be older and have lower CrCl values than patients in the other BMI categories, and these factors have previously been shown to be independently associated with increased risk of thromboembolism in Japanese patients [20, 21]. Furthermore, although CHADS_2_ scores were similar across the BMI categories, different patterns of comorbidities in patients who were underweight (i.e. a greater proportion with prior ischemic stroke/TIA compared with the other BMI groups) may have had an impact on thromboembolic outcomes. Indeed, analysis of the Fushimi AF Registry demonstrated that previous stroke/TIA and chronic kidney disease were strong independent risk factors for cardioembolic stroke in patients with AF even after adjustment for CHADS_2_ score components [22]. It should be noted that the proportion of patients who received an underdose of rivaroxaban in the present study was lowest (17.9%) in the underweight group and highest (32.5%) in the obese group (Supplementary Fig. 1); therefore, underdosing is unlikely to account for the increased incidence of thromboembolic events in patients who were underweight.

The finding from the present study that being underweight was independently associated with increased all-cause mortality is in agreement with results from studies conducted during the warfarin era [6] and the DOAC era [7, 23], and may be suggestive of a lean paradox rather than an obesity paradox [24]. Patients who are underweight tend to be susceptible to poor nutritional status [25], which may lead to becoming ill. Previous studies have concluded that increased mortality in patients who are underweight could, at least in part, be caused by residual confounding from pre-existing disease leading to weight loss [26]. Indeed, one hypothesis is that the obesity paradox in patients with AF may be due to confounding variables such as lead time bias. Patients who are underweight may be less likely to be tested for cardiovascular diseases than patients with obesity, and therefore, as a result, may receive a diagnosis only when the disease is more advanced, by which time prognosis is worse [27]. In particular, the current analysis revealed higher percentages of mortality from cancer, respiratory disorders, and infections in the underweight patient group compared to the other groups (Table 4). This result is consistent with a community-based cohort study targeting Japanese patients over 80 years old, in that mortality from cardiovascular disorders, cancer, or pneumonia tended to be highest in the underweight group compared with other groups, even though the differences did not reach statistical significance [28]. Those real-world data on causes of mortality in the underweight AF patients in the XAPASS study could be important information for physicians, because these multimorbid patients are often excluded in randomized clinical trials.

There are some limitations of this sub-analysis. First, BMI information was missing for 1960 patients (20.5%), which might have affected the results. Second, it is difficult to generalize the present findings to patients with NVAF receiving rivaroxaban worldwide, because the current study used doses approved in Japan (15 mg or 10 mg o.d.), which differ from doses used globally (20 mg or 15 mg o.d.). Also, the median BMI of the XAPASS (23.6 kg/m^2^) was lower than that of the ROCKET AF study (28.3 kg/m^2^). Third, the XAPASS is a single-arm, open-label observational study. It is impossible to directly compare the outcomes of rivaroxaban treatment with those of other treatments such as warfarin and other DOACs, as previously described [9]. Fourth, the sub-analysis included only 1 year of follow-up, limiting the ability to assess for late clinical events. Fifth, selection bias by physicians in prescribing rivaroxaban and loss of patients to follow-up might have led to underestimation of the event rates. Despite these limitations, we believe that our findings may help physicians make patient-specific decisions when prescribing rivaroxaban, especially for patients who are underweight or obese.

In this sub-analysis in Japanese patients with NVAF receiving rivaroxaban, BMI was not independently associated with major bleeding and thromboembolic outcomes, whereas it was independently associated with all-cause mortality. Given that this sub-analysis showed a high incidence of thromboembolic events and all-cause mortality in patients who were underweight, careful management of NVAF status and comorbidities may be required in this population.

## Electronic supplementary material

Below is the link to the electronic supplementary material.

Supplementary file1 (PDF 245 kb)

Supplementary file2 (PDF 182 kb)
