# Supplementary material for: Impact of body mass index on real-world outcomes of rivaroxaban treatment in Japanese patients with non-valvular atrial fibrillation
Source: Heart Vessels. 2020 Apr 6;35(8):1125–34. doi: 10.1007/s00380-020-01587-z (PMC7332477; doi:10.1007/s00380-020-01587-z)

Supplementary Figure 1. CrCl and rivaroxaban dosing in patients stratified by BMI*.

* BMI categories (kg/m^2): underweight, <18.5; normal, 18.5 to <25; overweight, 25 to <30; and obese, ≥30

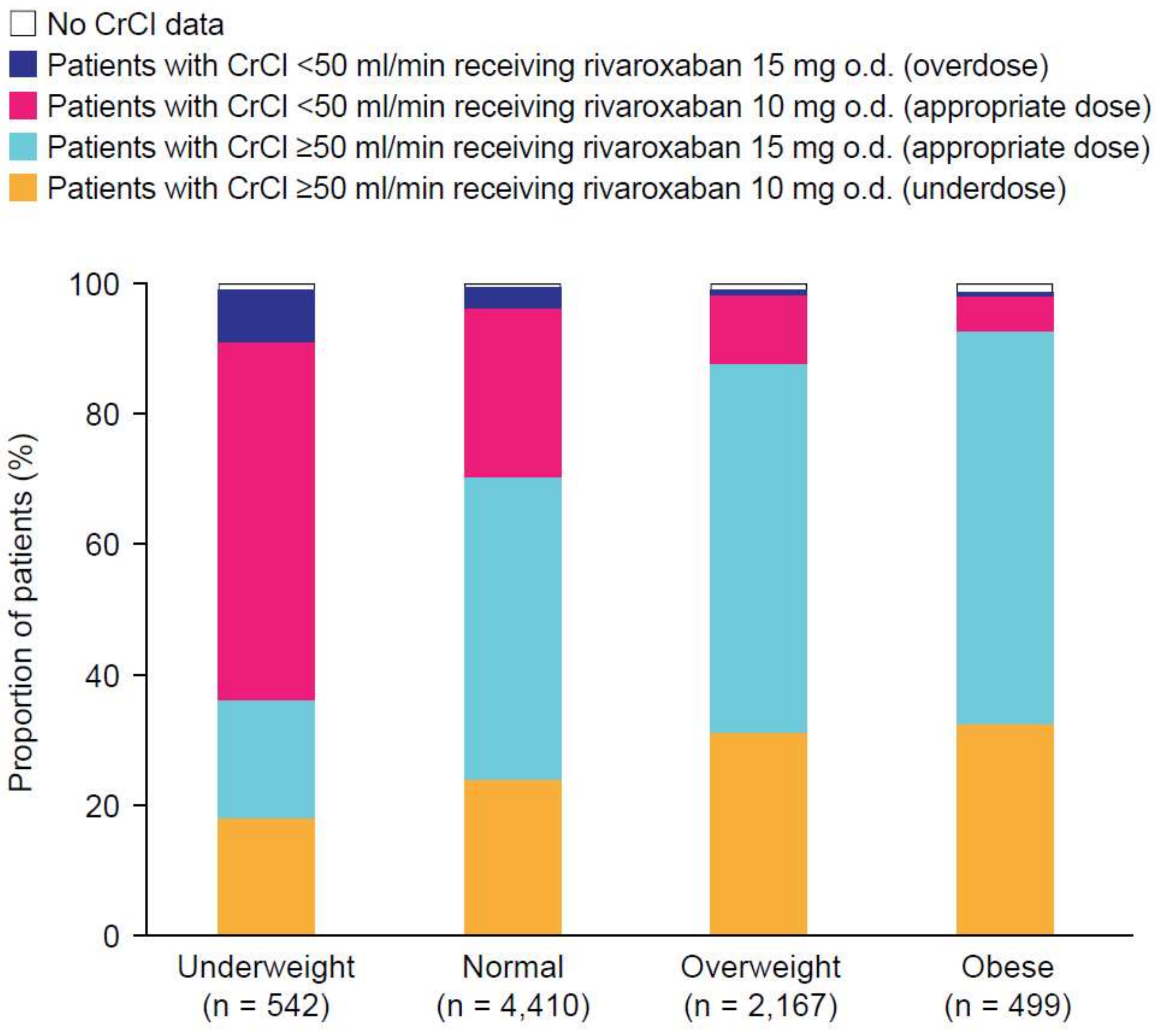

Supplement: Supplementary file 1 — Supplementary file1 (PDF 245 kb) [file 380_2020_1587_MOESM1_ESM.pdf]
