# Supplementary material for: Impact of body mass index on real-world outcomes of rivaroxaban treatment in Japanese patients with non-valvular atrial fibrillation
Source: Heart Vessels. 2020 Apr 6;35(8):1125–34. doi: 10.1007/s00380-020-01587-z (PMC7332477; doi:10.1007/s00380-020-01587-z)
Supplement: Supplementary file 2 — Supplementary file2 (PDF 182 kb) [file 380_2020_1587_MOESM2_ESM.pdf]

Supplementary Table 1

Safety and effectiveness outcomes

	BMI (kg/m²)			
	Underweight (<18.5)	Normal (18.5 to <25)	Overweight (25 to <30)	Obese (≥30)
Safety outcome	(n = 542)	(n = 4,410)	(n = 2,167)	(n = 499)
Any bleeding	46 (8.49)	307 (6.96)	128 (5.91)	23 (4.61)
Major bleeding	9 (1.66)	70 (1.59)	32 (1.48)	7 (1.40)
Fatal	1 (0.18)	4 (0.09)	6 (0.28)	0 (0)
Critical organ bleeding	3 (0.55)	32 (0.73)	18 (0.83)	0 (0)
Intracranial hemorrhage	3 (0.55)	29 (0.66)	15 (0.69)	0 (0)
Hemoglobin decrease ≥2 g/dl	3 (0.55)	26 (0.59)	10 (0.46)	5 (1.00)
Transfusion of ≥2 units of packed RBCs or whole blood	2 (0.37)	8 (0.18)	3 (0.14)	1 (0.20)
All-cause mortality	44 (8.12)	72 (1.63)	33 (1.52)	4 (0.80)
Death caused by adverse drug reaction	2 (0.37)	8 (0.18)	8 (0.37)	0 (0)
Effectiveness outcome	(n = 540)	(n = 4,392)	(n = 2,161)	(n = 497)
Stroke/non-CNS SE/MI	15 (2.78)	63 (1.43)	33 (1.53)	7 (1.41)
Stroke	12 (2.22)	58 (1.32)	30 (1.39)	5 (1.01)
Ischemic stroke	9 (1.67)	40 (0.91)	20 (0.93)	5 (1.01)

BMI = body mass index; CNS = central nervous system; MI = myocardial infarction; RBC = red blood cell; SE = systemic embolism.

Data are presented as n (%).
